# *BRCA1* regulates glucose and lipid metabolism in diabetes mellitus with metabolic dysfunction-associated steatotic liver disease via the PI3K/Akt signaling pathway

**DOI:** 10.1371/journal.pone.0318696

**Published:** 2025-03-26

**Authors:** Cui Ma, Xiaodi Yang, Liyin Zhang, Jie Zhang, Youyou Zhang, Xiaofeng Hu

**Affiliations:** 1 Department of Endocrinology, The First People’s Hospital of Yuhang District, Hangzhou, Zhejiang, China; 2 Department of Oncology, Minhang Branch, Zhongshan Hospital, Fudan University Shanghai, China, Key laboratory of whole-period monitoring and precise intervention of digestive cancer (SMHC), Minhang Hospital & AHS, Fudan University, Shanghai, China; 3 School of Sports Science and Engineering, East China University of Science and Technology, Shanghai, China; 4 Department of pharmacy, The First People’s Hospital of Yuhang District, Hangzhou, Zhejiang, China; 5 Department of Endocrinology, First People’s Hospital of Taizhou Affiliated Wenzhou Medical University, Taizhou, China; 6 Department of Cardiology, Shanghai Chest Hospital, Shanghai Jiao Tong University School of Medicine, Shanghai Jiao Tong University, Shanghai, China; Universite du Quebec a Montreal, CANADA

## Abstract

**Purpose:**

This study mimics the metabolic environment of metabolic dysfunction-associated steatotic liver disease (MASLD) and diabetic mellitus (DM) to investigate the function of *BRCA1* in regulating glucose and lipid metabolism in hepatocytes under high glucose (HG) settings.

**Methods:**

MASLD and DM-related datasets (GSE89632, GSE95849) were screened for overlapping genes, Protein-Protein Interaction (PPI) network and enrichment analyses were performed. Then, quantitative real-time polymerase chain reaction (qRT-PCR), Western Blotting (WB), and enzymatic colorimetric assays to examine the expression changes of *BRCA1* in mouse primary hepatocytes under HG conditions and the impact of the combined PI3K/Akt signaling pathway on key metabolic markers of gluconeogenesis and lipid metabolism.

**Results:**

Our study identified seven key overlapping genes (*AURKA*, *BRCA1*, *ISG15*, *NUSAP1*, *OAS1*, *RSAD2*, *TLR7*) between MASLD and DM. Experiments found that when *BRCA1* was overexpressed in mouse primary hepatocytes, intracellular triglyceride content and lipid metabolism-related biomarkers (such as PEPCK, SREBP-1c, G6Pase, and FAS) were significantly increased in HG circumstances. However, the knockdown of *BRCA1* reduced the expression of these indicators. Besides, we also observed that under HG conditions, the expression of proteins linked to the PI3K/Akt signaling pathway was negatively regulated by *BRCA1* expression. Moreover, TG content and expression of lipid metabolism markers are also regulated by *BRCA1* and PI3K/Akt pathway inhibitor Ly294002.

**Conclusion:**

As a key regulator of hepatocyte metabolism under HG conditions, *BRCA1* can participate in regulating glucose and lipid metabolism in mouse primary hepatocytes through the PI3K/AKT signaling pathway, which be able to become a possible remedy strategy for DM with MASLD.

## Introduction

Metabolic dysfunction-associated steatotic liver disease (MASLD) has become a growing public health problem worldwide. Globally, it is estimated that more than 25% of adults are affected by MASLD, and its prevalence has increased significantly over the past few decades [2].There is a strong association between MASLD and components of the metabolic syndrome, including obesity, hypertension, hyperglycemia, and hyperlipidemia [3]. Together, these metabolic abnormalities promote the accumulation of hepatic fat, which in turn may trigger an inflammatory response and hepatocellular injury, ultimately leading to hepatic fibrosis or even cirrhosis [4]. The pathogenesis of MASLD is complex and involves a variety of genetic, environmental and lifestyle factors. Although studies have revealed some key pathophysiological processes, including disturbances in hepatic lipid metabolism, oxidative stress, and alterations in intestinal flora, there are still many uncharted territories that need to be further explored [5]. Diagnosis of MASLD currently relies on liver imaging and liver biopsy, and the lack of specific biomarkers limits the early diagnosis and accurate assessment of MASLD [6]. Strategies for the treatment of MASLD currently focus on lifestyle changes, including dietary modifications, increased physical activity and weight management. Although some pharmacological therapies have shown potential efficacy, there is a lack of specific pharmacological treatments for MASLD. Therefore, the development of new therapeutic targets and strategies is essential to improve the prognosis of patients with MASL [7].

Diabetes mellitus (DM) combined with metabolic dysfunction-associated steatotic liver disease (MASLD) is a common metabolic disease characterized by the coexistence of diabetes and hepatic steatosis in the absence of heavy drinking [8,9]. In the context of MASLD and DM, insulin resistance does not equate to a complete shutdown of insulin signaling. In fact, a phenomenon known as “selective insulin resistance” occurs, where insulin signaling selectively activates certain metabolic pathways, such as de novo lipogenesis, without affecting others. This selective activation may lead to hepatic fat accumulation, thereby exacerbating the progression of MASLD [10–12]. Recent studies indicate that patients with both diabetes and MASLD were at a higher risk of developing progressive liver disease, cardiovascular complications, and liver-related mortality compared to those with either condition alone [13,14]. The pathogenesis of DM combined with MASLD is multifactorial and involves complex interactions among metabolic, genetic, and environmental factors [15]. Insulin resistance enhances the gluconeogenesis process in the liver and also promotes the synthesis of triglycerides [1] and fat [16,10]. These factors together lead to liver fat accumulation and aggravate the development of MASLD. Therefore, insulin resistance, which is common in patients with diabetes, is also a key link connecting these metabolic pathways. In addition, dyslipidemia, oxidative stress, and chronic low-grade inflammation are also key mechanisms leading to the occurrence and progression of this comorbidity [1,17]. The current focus of treating DM that is exacerbated by MASLD is on altering one’s diet and exercise routine to address the metabolic irregularities linked to both conditions [18,19]. New therapies are necessary, while the available alternatives for treatment are still restricted. Finding novel diagnostic biomarkers, therapeutic approaches, and prognostic indicators is crucial given the growing clinical importance and incidence of MASLD in DM.

*Breast cancer 1 gene* (*BRCA1*) is a tumour suppressor gene that plays an important role in DNA damage repair, cell cycle control and maintenance of genetic stability.Mutations in the BRCA1 gene are associated with an increased risk of hereditary breast and ovarian cancer [20]. In addition to controlling adipogenesis, *BRCA1* has also been shown to control the production of fatty acids in malignancies [21,22]. As shown in S1 Table, *BRCA1* is expressed in a variety of cell types, including hepatocytes, the primary focus of this study. This highlights a potential role for *BRCA1* in metabolic regulation. Research on the function of *BRCA1* in DM with MASLD has advanced significantly in recent years, offering new insights into the etiology and management of the condition. For instance, studies indicate that insulin resistance, a crucial aspect of diabetes, is caused by a *BRCA1* deficit [23]. Fatty liver disease develops in experimental animals with *BRCA1* mutations due to dysregulated lipid homeostasis and poor glucose metabolism. This study also showed a connection between inflammation and *BRCA1* when DM is present with MASLD. *BRCA1* deficiency increases proinflammatory cytokine production and leads to liver inflammation, which would be valuable to study in DM and MASLD.

The PI3K/Akt signaling system is an important signaling mechanism in cells related to hepatic glucose metabolism [24,25]. Studies have shown that the PI3K/Akt signaling system is critical for maintaining blood glucose homeostasis and plays a key role in insulin signaling [26]. In general, insulin stimulates PI3K to bind to its receptor and activate Akt [11,27]. By encouraging the production and translocation of glucose transporters like GLUT4, Akt activation enhances the uptake of glucose by muscle and adipose tissue [28]. Furthermore, Akt is involved in limiting the capability of the liver to produce glucose by inhibiting the gluconeogenesis process [29]. The hepatic insulin response is diminished when the PI3K/Akt signaling pathway is compromised or insulin resistance arises, which results in increased gluconeogenesis and aberrant blood sugar regulation [30]. This is a crucial factor in metabolic disorders including diabetes.

This study identified *BRCA1* as a hub gene through key overlapping gene analysis of MASLD and DM-related datasets. The effects of *BRCA1* overexpression and knockdown on glucose and lipid metabolism in mouse primary hepatocytes were then explored through *in vitro* cell experiments under hyperglycemic conditions (simulating the diabetic and MASLD environment). Following that, the complicated relationship between *BRCA1* expression, the PI3K/AKT signaling pathway, and dysregulation of glucose and lipid metabolism was investigated (S1 Fig). These findings may provide new clues for the study and treatment of the pathogenesis of DM complicated by MASLD.

## Materials and methods

### Differential gene expression analysis and visualization

We obtained the MASLD dataset (GSE89632) and the diabetes dataset (GSE95849) from the Gene Expression Omnibus (GEO, https://www.ncbi.nlm.nih.gov/geo). Among them, in the GSE89632 dataset, 39 samples (20 cases of simple steatosis and 19 cases of NASH) were regarded as the case group, and 24 healthy controls were used as the control group. The GS95849 dataset includes 18 samples, from which 6 diabetic samples (as the case group) and 6 healthy samples (as the control group) were selected for analysis. Next, using the R language’s Limma package, differentially expressed genes (DEGs) for each dataset were found. The fold change (FC) threshold criteria used were set to > 1.5 or < 0.67, with an adjusted *P* value of < 0.05. In addition, visualization was performed using the ggplot2 package of R software. Subsequently, the VennDiagram package was used to analyze overlapping genes in both datasets.

### Construction of key gene modules

The Search Tool for Retrieval of Interacting Genes (STRING; https://string-db.org/) for retrieving interacting genes was used to perform protein-protein interaction (PPI) analysis. The resultant PPI network was visualized using Cytoscape software, and its main modules were studied using three algorithms of the Cytohubba plug-in in the software, namely Maximum Neighbor Component (MNC), Maximum Clique Centrality (MCC) and Degree. Subsequently, the VennDiagram package was used again to identify overlapping genes in MNC, MCC, and Degree.

### Functional enrichment analysis and expression profiling of overlapping genes

For comprehensive functional annotation of overlapping genes and insights into their biological significance, we used the Database for Annotation, Visualization, and Integrated Discovery (DAVID; https://david.ncifcrf.gov/). Overlapping genes were submitted to the DAVID online platform, where enrichment analysis was performed for biological process (BP) and Kyoto Encyclopedia of Genes and Genomes (KEGG) pathways. The results were deemed statistically significant when *P* was less than 0.05. Next, to assess the expression patterns of the selected genes, we performed expression analysis using the GSE89632 and GSE95849 datasets. This enables us to investigate target gene expression patterns and levels in relation to NALFD and DM.

### Cell culture

Primary mouse hepatocytes were seeded in Dulbecco’s Modified Eagle’s Medium (DMEM) supplemented with 10% fetal bovine serum (FBS) and antibiotics from the BeNa Culture Collection (Beijing, China) [31]. We used primary hepatocytes, derived from C57BL/6 mice (Cell Biologics, USA). To promote hepatocyte health, the medium was supplemented with 1% insulin-transferrin-selenium (ITS) and 40 ng/mL dexamethasone. The cells were cultured in an incubator at 37°C with 5% CO_2_ that was humidified to offer optimal conditions for growth.

### Transfection and treatment assay

Primary mouse cells were transfected for overexpression and knockdown of *BRCA1*. Overexpression was promoted using a construct tagged over-*BRCA1*, whereas knockdown was achieved by two specific siRNAs (si-*BRCA1*#1 and si-*BRCA1*#2). Following transfection, high glucose (HG) was added to the cells at a concentration of 30 mM to replicate conventional *in vitro* settings with physiological glucose levels. Next, cells were treated with the PI3K/Akt pathway inhibitor LY294002 (supplementary concentration). To be sure the effects seen were caused by the inhibitor and not the solvent, a control group received an equivalent volume of DMSO treatment.

### Quantitative real-time polymerase chain reaction (qRT-PCR) assay

Total RNA was isolated from transfected primary mouse hepatocytes using a commercially available RNA extraction kit, such as Trizol reagent (Invitrogen, USA). A reverse transcription kit, such as the SuperScript IV First-Strand Synthesis System (Invitrogen, USA), was used to synthesize cDNA. The qRT-PCR was carried out with specific primers, the details of which are provided in Table 1. Finally, to find the associated factors’ expression levels, the 2^-ΔΔCt^ technique was employed.

**Table 1:** Primers sequence for qRT-PCR.

Target	Forward Primer	Reverse Primer
*BRCA1*	5′-AGTTGTGATCGTGCAGCCAAGC-3′	5′-ACAAGACGTGCCTTGCACAGCT-3′
G6Pase	5′-GCACATTTCCCTCACCAAGT-3′	5′-ACTTGGAGCCAGGTTGATG-3′
PEPCK	5′-CCGGGCACCTCAGTGAAG-3′	5′-CGTTGGTGAAGATGGTGTTTTTC-3′
SREBP-1c	5′-ATCGGCGCGGAAGCTGTCGGGGTAGCGTC-3′	5′-ACTGTCTTGGTTGTTGATGAGCTGGAGCAT-3′
FAS	5′-GTGCTGGAAAAGGAGACAGG-3′	5′-CCAGTGTCTGGGGTTGATTT-3′
β-actin	5′-AGGCCAANCGCGAAGAATGACC-3′	5′-GAAGTCCAGGGCGACGTAGGAC-3′

### Western blotting (WB) assay

Protease-inhibited RIPA lysis buffer was used to produce protein extracts from transfected primary mouse hepatocytes. The BCA protein assay kit (Thermo Fisher Scientific, USA) was utilized to ascertain the concentration of protein. Protein samples were separated by sodium dodecyl sulfate-polyacrylamide gel electrophoresis (SDS-PAGE) and transferred to polyvinylidene difluoride (PVDF) membranes. Following 5% non-fat milk blocking, primary antibodies against BRCA1 (1:1000), G6Pase (1:1000), ACCA (1:2000), PEPCK (1:1000), SREBP-1c (1:1000), FAS (1:1000), and β-actin (1:5000) as a loading control were treated with these membranes. Secondary antibodies conjugated to horseradish peroxidase were then incubated with the membranes following washing. An improved chemiluminescent detection technology, such as SuperSignal West Pico PLUS chemiluminescent substrate (Thermo Fisher Scientific, USA), was used to see protein bands.

### Enzyme colorimetric assay

To assess triglyceride concentrations, we employed an enzymatic colorimetric assay kit (Thermo DMA, Sigma). Samples were incubated with a triglyceride-specific enzyme that catalyzes the production of a colored product proportional to the triglyceride concentration. The reaction proceeded at 37°C and was terminated with a stop solution. Absorbance was measured spectrophotometrically, and triglyceride levels were deduced from a calibration curve. Each measurement was performed in triplicate to ensure accuracy. Blank wells, containing all reagents except the enzyme, served as controls to correct for non-specific color development. Results were normalized to protein content as quantified by the Bradford assay.

### Statistical analysis

With the aid of GraphPad Prism (version 9.0, GraphPad Software, USA), data were examined. Every experiment was conducted three times, with the results displayed as mean ±  SD. One-way ANOVA or Student’s t-test was used to determine statistical significance, and Tukey’s test was used for post hoc analysis. *P* <  0.05 was deemed significant.

## Results

### Identification of seven key overlapping genes in MASLD and DM related datasets

**Fig 1:**
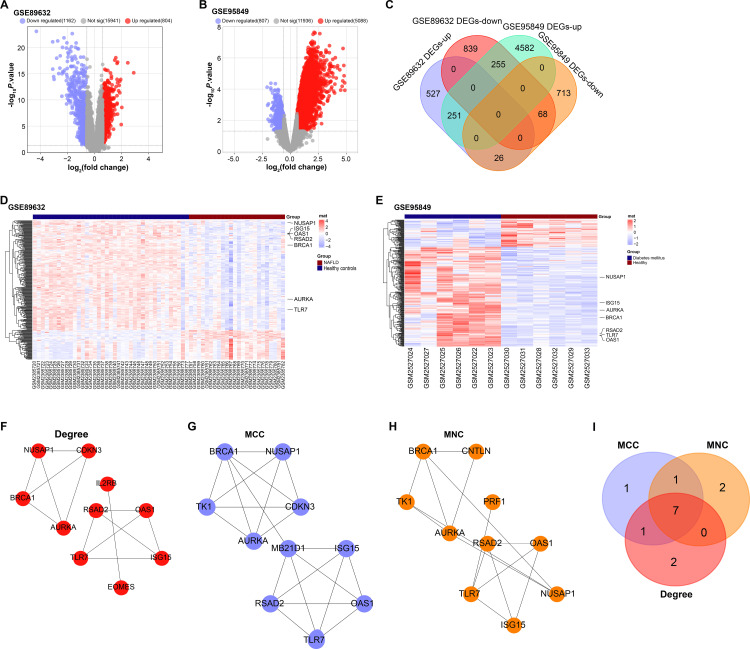
Screening and intersection analysis of MASLD and DM related DEGs. (A) Volcano plots, analysis of DEGs in GSE89632 dataset (A) and DEGs in GSE95849 dataset(B), red dots represent up-regulated DEGs, and green dots represent down-regulated DEGs. (C) Venn diagram showing overlapping analysis of DEGs in the GSE89632 and GSE95849 datasets. (D, E) Heatmap showing gene expression differences between the GSE89632 and GSE95849 datasets in the GEO database. Up-regulated genes are indicated in red and down-regulated genes are in blue, with the shade of color representing the magnitude of the change in expression. (F-H) Interaction network of constructed top 10 genes constructed by Degree(D), MCC(E), and MNC(F) algorithms. (I) Venn diagram, intersection analysis of key genes under MCC, MNC and Degree algorithms.

**Fig 2:**
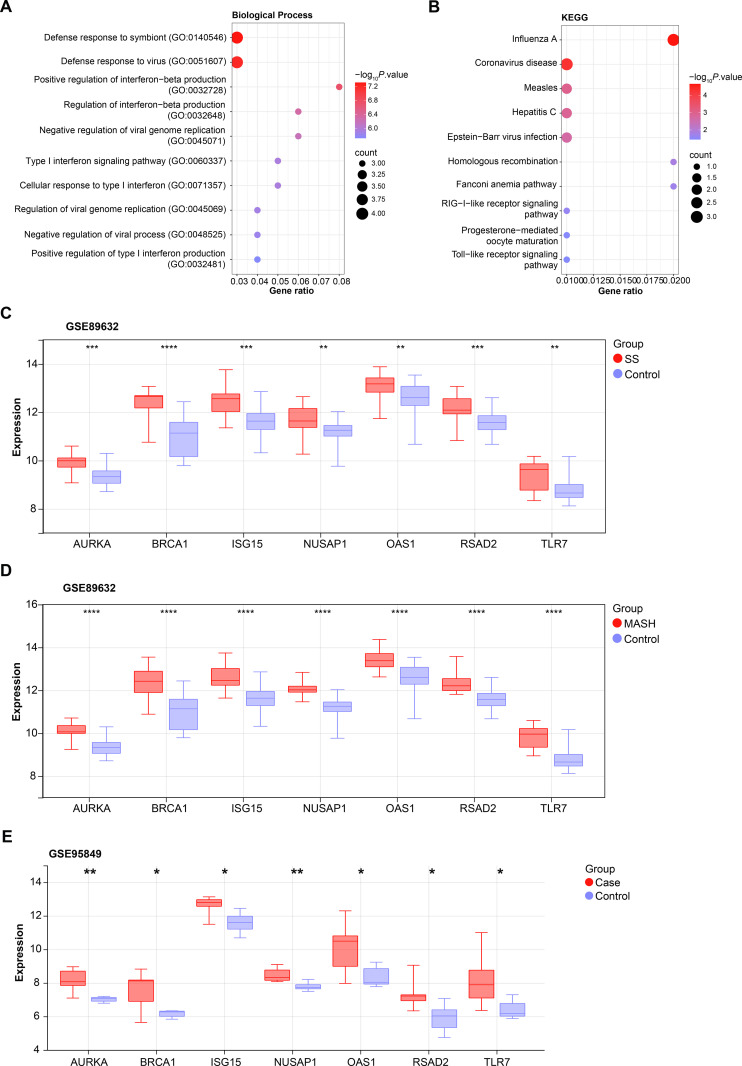
Functional pathway enrichment and expression analysis of seven overlapping genes. (A, B) Bubble plots of BP (A) and KEGG (B) enrichment analysis. The abscissa represents the proportion of genes, and the ordinate represents the top ten enrichment items. (C-E) Expression levels of seven genes in GSE95849 dataset and GSE89632 dataset. * *P* < 0.05, **P < 0.01, ***P < 0.001, ****P < 0.0001.

By analyzing two data sets (GSE89632 and GSE95849), we identified 1966 DEGs (804 up-regulated DEGs and 1162 down-regulated DEGs) related to MASLD, and 5895 DEGs (5088 up-regulated DEGs and 807 down-regulated DEGs) related to DM (Figs 1A, 1B). We then performed an overlap analysis on the DEGs identified in GSE89632 and GSE95849, from which we obtained 251 overlapping down-regulated DEGs and 68 overlapping down-regulated DEGs (Fig 1C). In both GEO datasets, heatmaps clearly demonstrate co-regulated genes (Fig 2D, 2E). In the GSE89632 dataset, the MASLD group showed significant expression changes in key genes such as NUSAP1, TLR7, AURKA, BRCA1, and RSAD2 compared to the healthy control group.The DM group in the GSE95849 dataset similarly showed expression differences in genes such as BRCA1, AURKA, ISG15, and NUSAP1. Then, PPI network analysis was performed on these genes, and three algorithms, MCC, MNC and Degree, respectively constructed the key modules of the top 10 genes (Figs 1F-1H). The intersection analysis of the three modules was performed again, and 7 key overlapping genes were obtained (Fig 1I). These seven genes are shown in S2 Table.

### Functional enrichment and expression analysis of 7 overlapping genes

To gain insight into the functional pathways associated with the seven overlapping genes, we performed pathway enrichment analysis using the clusterProfiler package. The results showed that overlapping genes were enriched in BP in the type I interferon signaling pathway and viral defense response (Fig 2A). In the KEGG pathway, these genes are related to the Hepatitis C, Toll-like receptor signaling pathway, and Fanconi anemia pathway (Fig 2B). Further expression analysis of these seven genes (*AURKA*, *BRCA1*, *ISG15*, *NUSAP1*, *OAS1*, *RSAD2*, *TLR7*) showed that their expression levels were generally higher in the experimental (simple steatosis [SS], metabolic dysfunction-associated steatohepatitis [MASH], Case) than in the control (Control) group in the GSE95849 and GSE89632 datasets (Fig 2C-2E). Their persistent overexpression raises the possibility that they have a role in the development and pathophysiology of DM and MASLD.

### *BRCA1* overexpression exacerbates HG-induced hepatocyte glucose and lipid metabolism disorders

**Fig 3:**
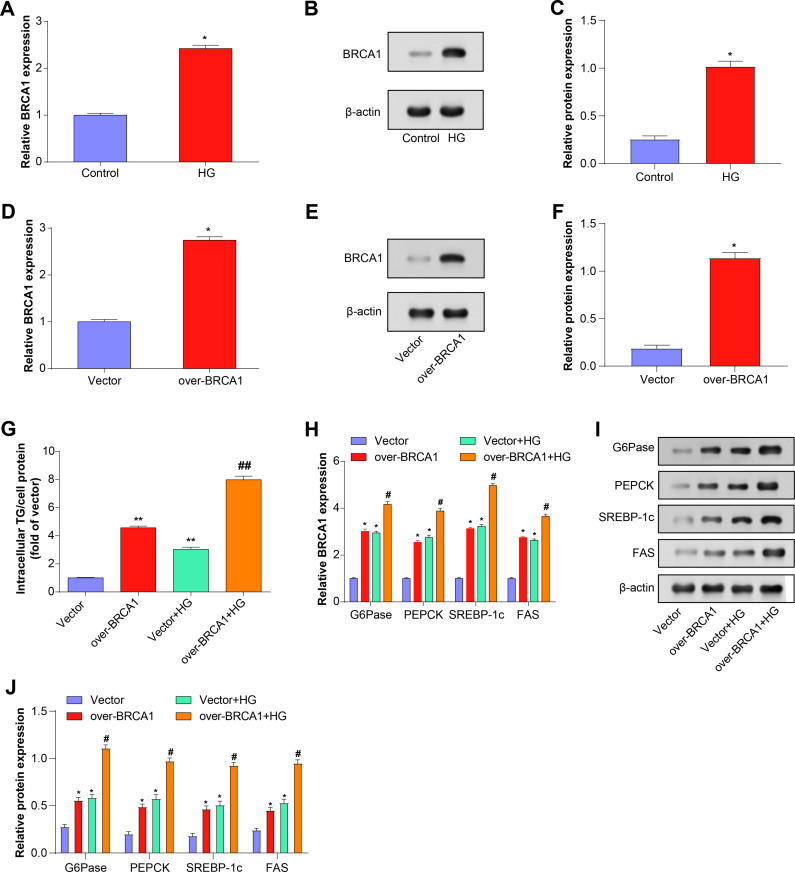
Effects of *BRCA1* overexpression on HG-induced TG and lipid metabolism in mouse hepatocytes. (A-C) *BRCA1* mRNA and protein expression levels in mouse primary hepatocytes under HG conditions were determined by qRT-PCR and WB. (D-F) The overexpression efficiency of *BRCA1* in mouse primary hepatocytes was determined by qRT-PCR and WB. (G) Enzymatic colorimetric assay to evaluate TG content in primary mouse hepatocytes after HG and *BRCA1* overexpression treatment. (H-G) After HG and *BRCA1* overexpression treatment, qRT-PCR and WB were used to determine the mRNA and protein expression levels of gluconeogenesis regulators and lipid synthesis regulators in mouse primary hepatocytes. * *P* < 0.05, **P < 0.01 compared to vector control; #*P* < 0.05, ##*P* < 0.01 compared to vector+HG treatment.

*BRCA1* mRNA and protein expression in primary mouse hepatocytes were significantly upregulated in response to HG exposure, as shown by qRT-PCR and WB analysis (Figs 3A-3C). Similarly, overexpression of *BRCA1* in mouse primary hepatocytes resulted in a significant increase in *BRCA1* levels, indicating significant overexpression transfection efficiency (Figs 3E-3F). The TG content in cells was assessed using an enzymatic colorimetric assay (Fig 3G). The results showed that the intracellular TG content increased significantly under HG conditions. Cells overexpressing *BRCA1* showed a further increase in TG content. Compared with the first two conditions, the TG content further increased after the simultaneous action of HG and over-*BRCA1*. PEPCK and G6Pase are key regulators of gluconeogenesis, whereas SREBP-1c and FAS are key regulators of lipid synthesis. Both *BRCA1* overexpression and HG therapy resulted in elevated degrees of PEPCK, FAS, G6Pase, and SREBP-1c in cells, according to qRT-PCR and WB analysis (Figs 3H-3J). The degrees of these metabolic indicators were most significantly elevated in cells in the combined *BRCA1* overexpression and HG state compared with the individual conditions.

### Hepatocytes with dysregulated glucose and lipid metabolism respond better to *BRCA1* knockdown

**Fig 4:**
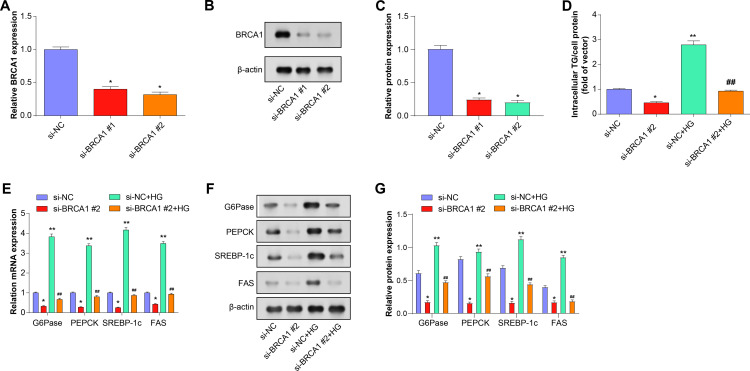
*BRCA1* knockdown alleviates dysregulation of glucose and lipid metabolism in mouse primary hepatocytes. (A-C) The mRNA and protein expression levels of *BRCA1* in primary mouse hepatocytes transfected with si-*BRCA1* #1 and si-*BRCA1* #2 were determined by qRT-PCR and WB. (D) Enzymatic colorimetric assay was used to evaluate the TG content in HG-induced mouse primary hepatocytes after *BRCA1* knockdown. (E-J) qRT-PCR and WB evaluated the expression of key regulatory factors of gluconeogenesis (PEPCK and G6Pase) and key regulatory factors of adipogenesis (SREBP-1c and FAS) in HG-induced mouse primary hepatocytes after knocking down *BRCA1*. * *P* < 0.05, **P < 0.01 compared to si-NC; #*P* < 0.05, ##*P* < 0.01 compared to si-NC+HG group.

To ensure the reliability and reproducibility of experimental results, we used multiple siRNA sequences for knockdown experiments and divided them into si-*BRCA1* #1 and si-*BRCA1*#2. Si-*BRCA1* #1 and si-*BRCA1* #2 transfected cells exhibited significantly lower *BRCA1* levels, with si-*BRCA1* #2 exhibiting a larger drop, according to qRT-PCR and WB analysis (Figs 4A-4C). Therefore, si-*BRCA1*#2 was selected for subsequent experiments. To further study the effects of *BRCA1* knockdown on metabolic dysregulation, we established si-NC, si-*BRCA1*#2, si-NC+HG, and si-*BRCA1*#2 + HG experimental groups. TG content in cells was assessed using an enzymatic colorimetric assay (Fig 4D). The findings demonstrated that, in comparison to controls, *BRCA1* knockdown alone significantly reduced TG content. After HG induction, intracellular TG content increased significantly. In addition, low-expression *BRCA1* reversed the increasing effect of HG on TG content, reaching a level comparable to that of the control group. qRT-PCR and WB analysis showed that knocking down *BRCA1* alone not only significantly reduced the expression of these markers. Moreover, *BRCA1* knockdown can also counteract the up-regulation effect on PEPCK, SREBP-1c, G6Pase, and FAS metabolic markers in HG circumstances (Figs 4E-4G).

### Knockdown or overexpression of *BRCA1* had no significant effect on ACCA expression.

Studies have suggested that *BRCA1* may influence the expression or activity of ACCA through the regulation of fatty acid metabolism, lipid synthesis, and other related pathways, thereby contributing to the modulation of fat metabolism. To explore this, we assessed the expression levels of BRCA1 and ACCA following *BRCA1* knockdown or overexpression. WB analysis revealed no significant changes in ACCA levels under either condition (S2A-C Fig). These findings indicate that, under the experimental conditions, *BRCA1* does not regulate the fatty acid metabolism pathway in hepatocytes through ACCA.

### *BRCA1* mediates the PI3K/AKT signaling pathway to affect glucose and lipid metabolism in mouse primary hepatocytes

**Fig 5:**
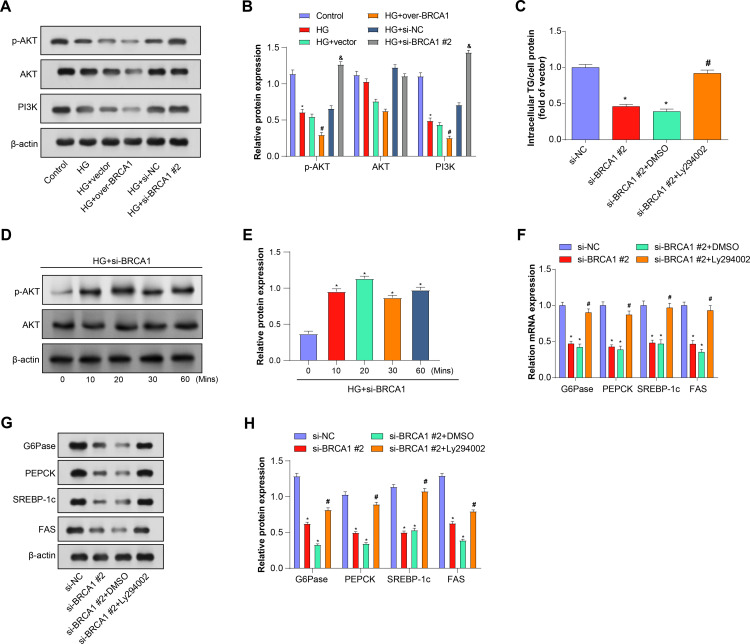
The regulatory effects of *BRCA1* and PI3K/Akt pathways on hepatic lipid metabolism under HG conditions. (A, B) WB evaluated the regulation of PI3K/AKT signaling pathway-related protein expression in primary mouse hepatocytes by changes in *BRCA1* expression before and after HG induction. (C) Enzymatic colorimetric determination of TG content in hepatocytes treated with *BRCA1* knockdown, DMSO, or PI3K inhibitor Ly294002. (D, E) WB examined the effect of knocking down BRCA1 at different time points on insulin-mediated AKT phosphorylation after induction by HG. (F-H) qRT-PCR and WB analysis shows the relative mRNA and protein expression levels of gluconeogenesis and lipogenesis genes (G6Pase, PEPCK, SREBP-1c, FAS) after different treatments. * *P* < 0.05 indicates a significant difference from si-NC, and #*P* < 0.05 indicates a significant difference from si-NC+DMSO group.

By assessing the expression levels and phosphorylation status of PI3K, p-AKT, and AKT in cell-based assays, the activation status of the PI3K/Akt signaling pathway and its role in cellular processes can be assessed. We examined the expression of PI3K/Akt pathway-related proteins in primary mouse hepatocytes before and after HG induction using WB analysis (Fig 5A-5B). The findings showed that under HG conditions, the expression of these proteins was downregulated. Expression levels were further reduced following HG induction in cells transfected with vector control. When *BRCA1* was overexpressed under HG conditions, the expression level was even lower. However, when si-NC was added, the protein level returned to that of the HG group. Likewise, protein levels in the si-*BRCA1#2* group were comparable to those in the control group. After HG induction, the TG content in mouse primary hepatocytes was assessed using an enzymatic colorimetric assay, as indicated by the trend shown in Fig 5C. The si-*BRCA1*#2 group showed decreased TG content, while the si-NC+DMSO group showed lower levels. Notably, when Ly294002 was added to the si-*BRCA1* #2 group, the TG content increased, almost reaching a level similar to that of the control group. We further explored the effect of *BRCA1* silencing on insulin-mediated AKT phosphorylation. By silencing *BRCA1* in primary mouse hepatocytes and using WB analysis at different time points, we examined the expression levels of p-AKT and AKT (Fig 5D-5E). The experimental results showed that under HG conditions, p-AKT levels were significantly higher in the *si*-*BRCA1*#*2* group compared with the control group at all time points, suggesting that the silencing of *BRCA1* may enhance the promotion of AKT phosphorylation by insulin. Notably, although p-AKT levels were elevated in the *si*-*BRCA1*#*2* group, this elevation did not show a significant time-dependent increase with time, suggesting that the effect of *BRCA1* silencing on AKT phosphorylation may have reached its maximum effect at an early stage. HG-induced expression levels of key regulators of gluconeogenesis (PEPCK and G6Pase) and lipid synthesis (SREBP-1c and FAS) in primary mouse hepatocytes were assessed using qRT-PCR and WB (Figs 5F-5H). Expression of these regulators was decreased in the si-*BRCA1* #2 group. Notably, in the si-NC+DMSO group, the expression level continued to decrease. Regulator expression levels in the si-*BRCA1* #2 + Ly294002 group were restored and almost reached levels comparable to those in the control group. These results implied that *BRCA1* regulation impacts metabolic indicators in primary mouse hepatocytes as well as the activity of the PI3K/Akt signaling pathway.

### siRNA technology was used to explore the role of BRCA1 in regulating glycolipid metabolism through PI3K/Akt signaling pathway in MASLD

**Fig 6:**
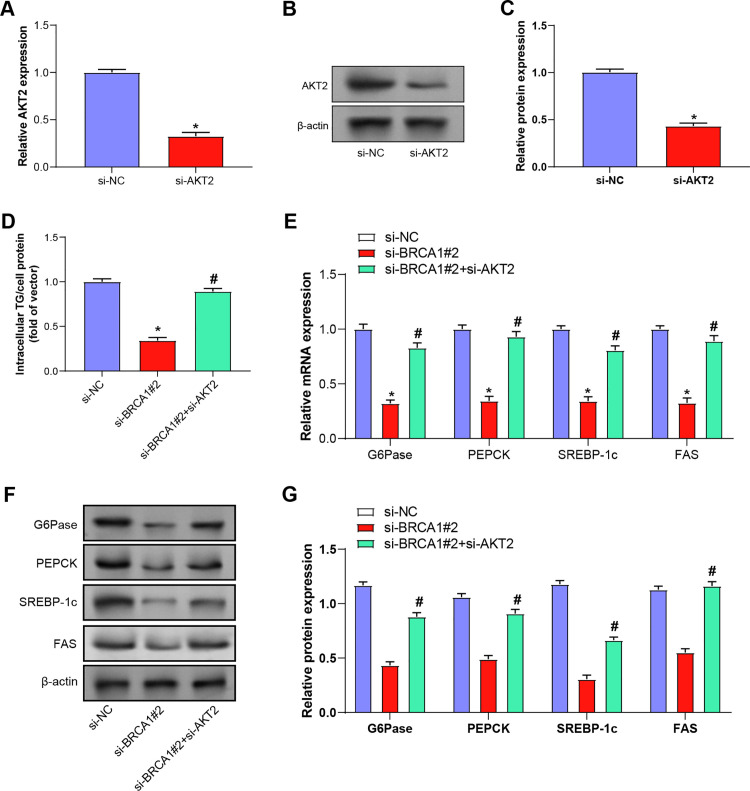
siRNA technology was used to explore the role of *BRCA1* in regulating glycolipid metabolism through PI3K/Akt signaling pathway in MASLD. (A-C) qPCR and WB were used to assess the efficiency of *AKT2* gene knockdown. (D) The TG content in hepatocytes treated with BRCA1 knockdown and simultaneous *BRCA1* and *AKT2* knockdown was determined by enzymatic colorimetric assay. (F-G) qRT-PCR and WB analysis shows the relative mRNA and protein expression levels of gluconeogenesis and lipogenesis genes (G6Pase, PEPCK, SREBP-1c, FAS) after different treatments. * *P* <  0.05 indicates a significant difference compared to the si-NC group, and #*P* <  0.05 indicates a significant difference compared to the *si-BRCA1 #2* group.

SiRNA was used to silence key genes in the PI3K/Akt signaling pathway to study the role of the signaling pathway in regulating lipid metabolism. We paid particular attention to the *AKT2* gene because it may play an important role in MASLD. Through siRNA-mediated silencing of the *AKT2* gene, we observed changes in lipid metabolism-related protein and mRNA expression. In mouse primary hepatocytes, we observed a significant decrease in the expression level of *AKT2* protein after silencing the AKT2 gene (*si-AKT2*) by siRNA technology (Fig 6A-6C). This suggests that siRNA-mediated gene silencing is effective. Simultaneous silencing of *BRCA1* and *AKT2* (*si-BRCA1#2* + *si-AKT2*) resulted in a significant increase in intracellular TG content compared with silencing BRCA1 alone, which was similar to that of drug inhibitor LY294002 (Fig 6D). After silencing *BRCA1* (*si-BRCA1#2*) or silencing both BRCA1 and *AKT2* (*si-BRCA1#2* + *si-AKT2*), we measured mRNA expression levels of several key genes involved in lipid metabolism (Fig 6E). The results showed that the mRNA expression levels of *PEPCK*, *G6Pase*, *SREBP*-*1c* and *FAS* were significantly reduced after *BRCA1* silencing, and si-AKT2 further enhanced this effect. These results suggest that *BRCA1* may positively regulate the expression of these genes through the PI3K/Akt signaling pathway. At the protein level, we also observed similar changes (Fig 6F and 6G). The important role of *BRCA1* and *AKT2* in the regulation of lipid metabolism was further confirmed. These results support the hypothesis that *BRCA1* mediates the PI3K/AKT signaling pathway, affecting glucose and lipid metabolism in mouse primary hepatocytes.

## Discussion

The prognosis of DM with MASLD is complicated, and it depends on several variables, such as the severity of the illness, coexisting conditions, and responsiveness to therapy [32]. MASLD, which ranges from simple fatty liver to non-alcoholic steatohepatitis (NASH), is currently the most common liver disease worldwide and is strongly associated with obesity, insulin resistance and type 2 diabetes mellitus (T2DM). Notably, while NASH can lead to serious liver diseases such as cirrhosis and hepatocellular carcinoma, most patients with NASH ultimately die from complications related to T2DM and cardiovascular disease [33]. MASLD and T2DM often coexist and synergistically increase the risk of adverse intra- and extrahepatic clinical outcomes. T2DM is one of the strongest risk factors for progression of MASLD to NASH, progressive fibrosis, or cirrhosis. Interestingly, the relationship between MASLD and T2DM is bidirectional and complex [34]. Epidemiological studies have shown that T2DM doubles the odds of MASLD and vice versa, mainly mediated by insulin resistance (IR). In addition, MASLD increases the risk of developing T2DM approximately twofold regardless of the presence or absence of common metabolic risk factors such as obesity, and rises further with increasing severity of liver fibrosis [35]. For efficient treatment of diabetes and MASLD, an accurate and prompt diagnosis is essential. These days, a variety of clinical factors are used to make the diagnosis, such as the patient’s medical history, physical exam results, liver enzyme testing, and imaging methods including magnetic resonance imaging and ultrasound [36,37]. These diagnostic techniques’ specificity, sensitivity, and capacity to distinguish between various disease stages are all limited. Targeted gene therapy attempts to delay the progression of liver pathology and metabolic diseases by regulating certain genes implicated in these conditions [38]. This restores normal cellular function. A few genes have been discovered as possible targets for MASLD gene therapy, including SREBP-1c and PPARα [39]. To improve gene delivery techniques, examine long-term safety, and determine if these therapies are therapeutically effective, more study is necessary.

T2DM-MASLD is the fourth leading cause of death. Hyperglycaemia can lead to a variety of complications, including kidney disease, cirrhosis of the liver, and ultimately may lead to hepatocellular carcinoma (HCC). Liver disease develops from a variety of etiological factors, including insulin resistance and oxidative stress. Free fatty acid (FFA) accumulation in the liver causes oxidative stress and endoplasmic reticulum (ER) stress. Hepatocellular injury induces the release of inflammatory cytokines from Kupffer cells (KC), which further activate hepatic stellate cells (HSC) [40]. Seven overlapping genes were removed from the DM and MASLD-related data sets for our investigation, and enrichment analysis was carried out. Among them, it has been established that the hepatitis C virus and the Toll-like receptor signaling pathway are connected to the pathophysiology of DM and MASLD. For example, an increased risk of T2D and insulin resistance (IR), which results in liver illnesses like MASLD, is linked to hepatitis C virus (HCV) infection [41]. This correlation emphasizes how insulin resistance, a contributing factor to the development of diabetic nephropathy, and diabetic inflammatory responses are mediated by TLR signaling. Furthermore, it has been demonstrated by previous research that TLR signaling pathways that are triggered by changes in the gut microbiota and its metabolites are crucial to the pathophysiology of metabolic dysfunction-associated steatotic liver disease [42]. The TLR4 signaling pathway may be used by conventional treatments like Dahuang Alistie Decoction to treat MASLD. These findings suggest that the above pathways may have a role in the development and pathophysiology of DM and MASLD, and this steady increase supports the theory that these genes are important for the biological processes of comorbidity.

Among the seven genes related to DM and MASLD, we selected *BRCA1* as the hub gene for analysis. Previous studies have shown that *BRCA1* is involved in cell cycle regulation, DNA damage repair, and maintenance of genome stability [43,44]. As described by Bruand M et al., *BRCA1* loss leads to transcriptional reprogramming of ovarian cancer cells [45]. Zhu Q et al. Reports indicate that the *BRCA1*-BARD1 heterodimer functions through multiple steps during homologous recombination (HR) to ensure rapid repair of DNA double-strand breaks [46]. Wang He et al. Loss of *BRCA1* function in luminal MECs was found to promote the development of basal-like breast tumors [47]. The results showed that knockdown of BRAP reduced the proliferation and migration of gastric cancer cells and inhibited the epithelial-mesenchymal transition (EMT) process. Overexpression of BRAP induced the proliferation, migration and EMT process of gastric cancer cells [48]. Radiotherapy significantly reduced cell viability and colony forming ability of *BRCA1* knockdown cells compared to control cells. These results suggest that *BRCA1* knockdown enhances the sensitivity of MDA-MB231 breast cancer cells to radiotherapy [49]. TGF-β1 knockdown increases chemosensitivity by promoting *BRCA1* expression and Smad3 phosphorylation. In vivo studies showed that TGF-β1 knockdown significantly inhibited tumor growth, and this effect was achieved by upregulating *BRCA1* expression and Smad3 phosphorylation [50]. These findings highlight the complex roles that *BRCA1* plays in a variety of cellular processes such as DNA repair, cancer cell proliferation, and therapeutic response. Additional studies on the mechanism of action of *BRCA1* through *in vitro* cellular studies may provide new perspectives for patient management and clinical diagnosis. Thus, further elucidation of the multiple functions of *BRCA1* could pave the way for novel therapeutic strategies. This demonstrates the contribution of *BRCA1* to genome stability and metabolic regulation, allowing us to understand its multifaceted functions. We hypothesized that *BRCA1* may be connected to the development of these two illnesses after finding that it was considerably highly expressed in both DM and MASLD samples. More studies into the mechanism of action of *BRCA1* employing *in vitro* cell investigations may thus give new views on patient management and clinical diagnostics.

MASLD, lipid metabolism, and diabetes are all strongly correlated with two key metabolic processes in humans: lipogenesis and glucose synthesis [51]. Changes in lipogenesis may also be linked to the onset of diabetes, as the liver’s gluconeogenesis pathway may be unusually active and cause hyperglycemia [52]. Increased lipogenesis is one of the primary reasons for the fat buildup in the liver, which is linked to the onset of MASLD [53]. PEPCK and G6Pase are two important regulators of gluconeogenesis, which is the process by which non-carbohydrates are converted to glucose [54]. FAS and SREBP-1c are two important adipogenesis regulators that are particularly important for the occurrence and progression of metabolic diseases such as diabetes and MASLD [55]. They also play an important role in maintaining blood glucose levels and energy balance in humans. Furthermore, TG is not only an important aspect of lipogenesis but also plays a crucial role in MASLD, and its overproduction can cause liver damage and exacerbate MASLD [56]. Therefore, controlling TG levels and the balance of gluconeogenesis and adipogenesis is crucial to managing and avoid diabetes and MASLD. We induced HG in primary mouse hepatocytes and found that *BRCA1* overexpression increased the levels of PEPCK, G6Pase, SREBP-1c, and FAS, while *BRCA1* knockout had the opposite effect. This suggests that *BRCA1* may be involved in regulating lipid metabolism in conditions similar to diabetes.

Phosphatidylinositol in cells is derived from RTKs and is associated with the PI3K-Akt signaling pathway [57]. Research shows it is critical for insulin stimulation and cell survival [58]. Among these, by contributing to insulin resistance, PI3K/Akt signaling creates a feedback loop in the development of obesity and type 2 diabetes, and its failure can aggravate both metabolic diseases [24]. Through its regulation of cell survival, proliferation, and metabolism, the PI3K/Akt signaling pathway can also impact the course and management of diabetes. According to another study, Apelin-13 activates the PI3K/Akt pathway in gestational diabetes (GDM) mice, improving glucose and lipid metabolism and reducing damage from oxidative stress and inflammatory response [59]. Moreover, Camaya I et al. found out that the PI3K/Akt signaling pathway can help tackle issues related to diabetes [60]. It’s crucial for keeping pancreatic β-cell survival and function in check. On the other hand, this pathway plays a role in inflammation, fibrosis, and hepatic steatosis. These factors can lead to nonalcoholic steatohepatitis (NASH), hepatocellular carcinoma, and MASLD. Supporting this idea, Matsuda S and the team highlighted in their research that the PI3K/Akt signaling pathway is key in the transition from a basic fatty liver to steatohepatitis and liver fibrosis [61]. This prompted us to investigate how *BRCA1* and the PI3K/Akt signaling pathway regulate lipid metabolism in primary mouse hepatocytes. Our results indicate that there is a close relationship between changes in *BRCA1* expression and important lipid metabolism indicators as well as PI3K/AKT pathway-related proteins. In essence, our study uncovers potential new targets for treating conditions like diabetes mellitus and MASLD. It underscores the significance of the *BRCA1*-mediated PI3K/Akt signaling pathway in the development and progression of metabolic disorders.

In our study, we observed downregulation of *BRCA1* expression in MASLD, which may be associated with disease progression. Although our data do not directly explain this phenomenon, according to the existing literature, *BRCA1* expression may be regulated by a variety of factors, including epigenetic modifications, the influence of microRNAs, and changes in the activity of transcription factors. For example, it has been suggested that miR-146a and miR-146b-5p may bind to and downregulate the expression of BRCA1 by binding to the same site in its 3’UTR [62]. Downregulation of BRCA1 may be associated with alterations in multiple biological processes, including cell cycle control, impairment of DNA repair mechanisms, and disruption of cellular metabolism. These changes may collectively contribute to the development and progression of MASLD. In addition, downregulation of *BRCA1* expression may also be associated with activation of other metabolic pathways, which needs to be further explored in future studies. Although the current study did not directly reveal the specific mechanism of BRCA1 expression downregulation, our findings provide an important foundation for future studies. Future studies can make use of high-throughput sequencing technology, gene editing tools such as CRISPR-Cas9, and bioinformatics analyses to explore in depth the mechanism of *BRCA1*’s role in MASLD. These studies will contribute to a better understanding of the molecular pathology of MASLD and may reveal new therapeutic targets.

Despite limited treatment options for non-alcoholic steatohepatitis (MASLD), recent research advances offer new hope for patients. Of particular note, since March 2024, the U.S. Food and Drug Administration (FDA) has approved Resmetirom for the treatment of MASLD. Resmetirom is a new therapeutic agent whose mechanism of action is to promote hepatic fatty acid oxidation and energy expenditure through activation of thyroid hormone β-receptor (TRβ), while inhibiting fat accumulation and inflammatory response in the live [63]. The approval of Resmetirom marks a major breakthrough in the treatment strategy for MASLD, providing patients with a potential non-invasive therapeutic option. Clinical trial data have shown that Resmetirom significantly improves liver fat content, reduces liver inflammation, and improves liver function in patients with MASL [64]. In addition, Resmetirom has relatively few side effects, making it an attractive treatment option in the management of patients with MASLD. However, while the approval of Resmetirom provides a new option for the treatment of MASLD, further studies are needed to determine its long-term efficacy and safety, as well as its applicability in different MASLD subtypes. Future studies should focus on the effectiveness of Resmetirom in combination with other treatments (e.g., lifestyle interventions, pharmacological treatments) and its potential role in preventing the progression of MASLD to more serious liver diseases such as cirrhosis and hepatocellular carcinoma. In summary, the approval of Resmetirom provides a new perspective on the treatment of MASLD, emphasising the importance of targeted therapies that address the pathophysiological mechanisms of the disease. As more innovative drugs are developed and approved, we expect that treatment strategies for MASLD will become more diverse and personalised to better meet the needs of patients.

## Limitation

Despite the valuable insights gained from our study, it is important to acknowledge that there are several limitations that may affect the interpretation and generalisation of our findings. First, we did not directly validate the predicted protein-protein interactions (PPIs) in our experiments. Although bioinformatics analyses provided valuable preliminary data, these results need to be further validated by experimental methods such as co-immunoprecipitation (Co-IP) and mass spectrometry. Due to technical limitations, we currently do not have direct access to cell type-specific expression data for these candidate proteins. We recognise that this is an important area of research and we acknowledge that the information we have provided is incomplete. Second, we mainly focused on the overexpression status of BRCA1 under high-glucose conditions, without delving into the status of the PI3K/Akt pathway and its relationship with BRCA1 under normoglycemic conditions, which needs to be further analysed and explored in future studies. Then, our experimental design was limited to specific tissues and failed to directly investigate the effects of changes in BRCA1 expression levels on other tissues. Finally, our study was mainly based on an in vitro experimental model and lacked mechanism validation under in vivo conditions. Therefore, future studies will include experimental validation of PPI while exploring the cell type-specific expression of these candidate proteins and analyses of the state of the PI3K/Akt pathway under normoglycaemic conditions, explorations of the interaction of BRCA1 with this pathway. We suggest that future studies should also include in vivo experiments in mouse models to validate our findings and explore in depth the role of BRCA1 in the development of MASLD and DM. These experiments will contribute to a more accurate understanding of the function of BRCA1 in metabolic regulation and its potential as a potential therapeutic target. In addition, we plan to utilize the GEO dataset of liver-specific PTEN knockout (PTENKO) mice (e.g., GSE70681), a mouse model with constitutive AKT overactivation, in our follow-up studies, which will provide us with valuable information to complement our current in vitro findings. Through these extended studies, we hope to provide deeper biological insights and advance the field.

## Conclusion

To sum up, we discovered that *BRCA1* influences the processing of fat and glucose by mouse hepatocytes and plays a crucial role in regulating the PI3K/Akt signaling pathway. Our in-depth research shows that when *BRCA1* is overexpressed, it cranks up important metabolic signals like FAS, PEPCK, G6Pase, and SREBP-1c. This can lead to elevated triglyceride levels and worsen metabolic problems caused by excess glucose. However, the knockdown of *BRCA1* successfully resolved these metabolic issues. This suggested that *BRCA1* may be an effective target for treating diseases such as diabetes and metabolic dysfunction-associated steatotic liver disease. Our findings shed light on the complicated processes of metabolic control and emphasize the function of the *BRCA1*-mediated PI3K/AKT signaling pathway in the etiology and progression of metabolic disorders. These results not only add to our understanding of cellular metabolic processes but also pave the way for new treatment approaches to metabolic illnesses.

## Supporting information

S1 Fig The mechanism of action of *BRCA1* in regulating glucose and lipid metabolism in DM complicated by MASLD patients.
Under the HG environment, *BRCA1* participates in regulating the glucose and lipid metabolism of hepatocytes through the PI3K/Akt pathway.
(TIF)

S2 Fig Knockdown or overexpression of *BRCA1* had no significant effect on ACCA expression.
(A-C) qRT-PCR and WB analysis showed the relative expression levels of BRCA1 and ACCA mRNA and protein after knockdown or overexpression of *BRCA1*. * *P* <  0.05 indicates significant difference from si-NC or Vector group.
(TIF)

S1 Table Single-cell analysis of expression levels of seven key overlapping genes in different cell types.
Each row in the table represents a specific cell type, listing the number of cells in that cell type and the expression level of each gene. *BRCA1* has the highest expression level in hepatocytes (Hepatocytes, Liver).
(XLSX)

S2 Table Expression of seven key overlapping genes in the GSE95849 dataset.
The table lists the expression of seven key overlapping genes (*AURKA*, *BRCA1*, *ISG15*, *NUSAP1*, *OAS1*, *RSAD2*, *TLR7*) in diabetic patients and healthy individuals in the GSE95849 dataset (expressed in logFC), as well as the relevant *P* values. Among them, *AURKA* and *BRCA1* are highly significantly correlated with the risk of diabetes.
(XLSX)
