# Supplementary figures and images for: BRCA1 regulates glucose and lipid metabolism in diabetes mellitus with metabolic dysfunction-associated steatotic liver disease via the PI3K/Akt signaling pathway
Source: PLoS One. 2025 Mar 26;20(3):e0318696. doi: 10.1371/journal.pone.0318696 (PMC11940781; doi:10.1371/journal.pone.0318696)

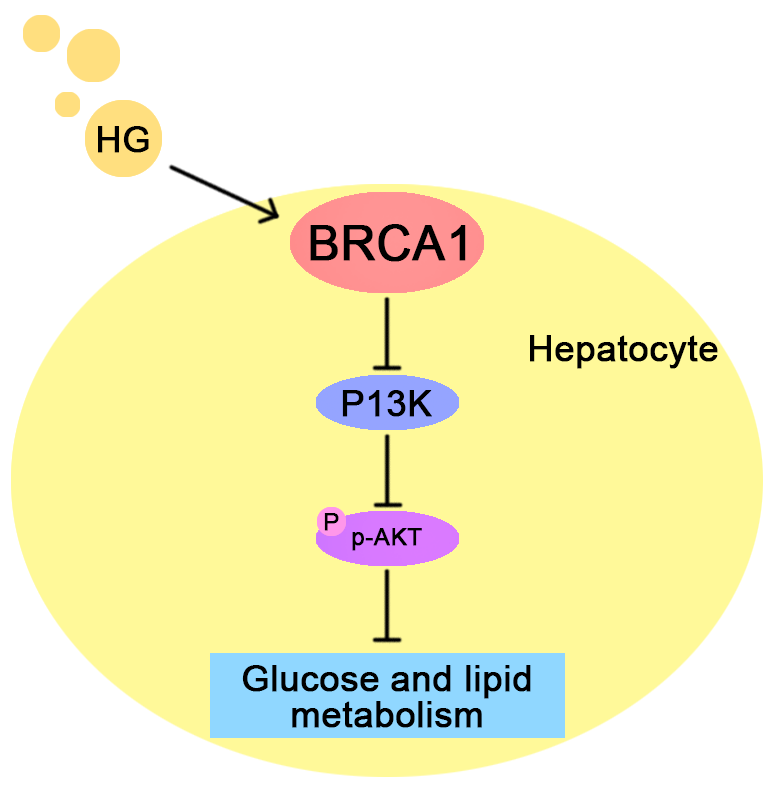

Supplement: S1 Fig — Under the HG environment, BRCA1 participates in regulating the glucose and lipid metabolism of hepatocytes through the PI3K/Akt pathway. (TIF) [file pone.0318696.s001.tif]

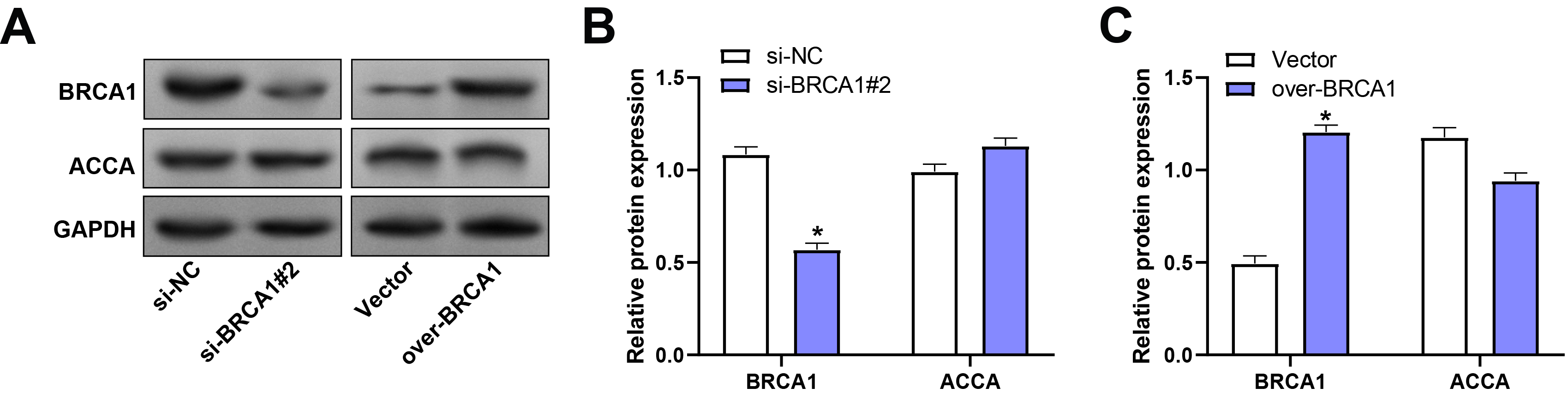

Supplement: S2 Fig — (A-C) qRT-PCR and WB analysis showed the relative expression levels of BRCA1 and ACCA mRNA and protein after knockdown or overexpression of BRCA1. * P < 0.05 indicates significant difference from si-NC or Vector group. (TIF) [file pone.0318696.s002.tif]
